# One-Stitch Versus Traditional Ileostomy After Low Anterior Resection for Rectal Cancer: A Retrospective Cohort Study

**DOI:** 10.3390/medicina62030423

**Published:** 2026-02-24

**Authors:** Ahmet Sencer Ergin, Ali Karabulut, Alparslan Saylar, Nihat Buğdaycı, Hakan Yiğitbaş

**Affiliations:** Department of General Surgery, Bağcılar Training and Research Hospital, Istanbul 34200, Turkey; alikarabulut7676@gmail.com (A.K.); saylaralparslan@gmail.com (A.S.); nihatbugdayci@gmail.com (N.B.); drhyigitbas@yahoo.com.tr (H.Y.)

**Keywords:** rectal cancer, low anterior resection, diverting ileostomy, OM, surgical outcomes

## Abstract

*Background and Objectives*: Diverting ileostomy is frequently used after low anterior resection (LAR) for rectal cancer to mitigate the clinical consequences of anastomotic leakage. The one-stitch method (OM) has been proposed as a simplified alternative to the traditional method (TM), with potential procedural advantages. However, evidence regarding its short-term outcomes and procedural efficiency remains limited and largely context-specific. This study aimed to compare perioperative outcomes of OM and TM in a single-center cohort. *Materials and Methods*: This retrospective cohort study included patients who underwent LAR with diverting ileostomy for rectal cancer, between January 2022 and November 2025. A total of 67 patients were analyzed (OM: *n* = 31; TM: *n* = 36). Operative time, intraoperative blood loss, length of hospital stay, stoma-related complications, overall postoperative morbidity and anastomotic leakage were compared. Subgroup analysis was performed for laparoscopic cases. Multivariable logistic regression was used to explore factors associated with postoperative complications. *Results*: Baseline demographic and clinical characteristics did not differ significantly between groups. The OM was associated with shorter operative time and lower intraoperative blood loss compared with TM, both in the overall cohort and in the laparoscopic-only subgroup. No statistically significant differences were observed between OM and TM regarding stoma-related complications, overall postoperative complications or anastomotic leakage. Length of hospital stay was shorter in the TM group. In multivariable analysis, ileostomy technique was not independently associated with postoperative complications, whereas laparoscopic surgery was associated with a lower likelihood of postoperative complications. Given the limited sample size, the study was underpowered for infrequent safety endpoints. *Conclusions*: In this single-center retrospective analysis, the OM was associated with improved procedural efficiency but did not demonstrate a clear advantage in postoperative recovery or hospital stay. No statistically significant differences in short-term morbidity were observed; however, equivalence cannot be inferred due to limited statistical power. These findings should be interpreted as regional validation data and underscore the need for larger prospective studies incorporating longer-term and patient-centered outcomes.

## 1. Introduction

Colorectal cancer is the third most common type of cancer in the world and the second leading cause of cancer-related death [1]. Although various treatment methods have been described, radical surgery remains one of the most significant treatment methods for this type of cancer. With the development of minimally invasive techniques in recent years, laparoscopic low anterior resection (LAR) has become the most frequently performed procedure for rectal cancer [2].

In rectal cancer surgery, whether performed through open or minimally invasive methods, one of the most significant complications is anastomotic leakage. This condition represents a significant cause of morbidity and mortality, as well as a leading cause of decreased oncological treatment success and prolonged hospital stay [3,4,5,6,7]. Studies have shown that creating a diverting ileostomy reduces complications associated with anastomotic leaks [8,9,10]. However, stoma-related complications such as bleeding, stenosis, prolapse, parastomal hernia, mucocutaneous dehiscence, as well asmetabolic problems due to high flow rates, both reduce the patient’s quality of life and increase stoma care costs [11,12,13,14]. For these reasons, choosing the appropriate diverting ostomy is extremely significant.

The traditional method (TM) of loop ileostomy involves fixation of the bowel to the abdominal wall using multiple sutures at the level of the peritoneum, fascia and skin. Although widely practiced, this technique is relatively time-consuming and may contribute to local tissue trauma. In contrast, the one-stitch method (OM) has been proposed as a simplified alternative, relying on a single fixation suture passed through the mesenteric avascular area to secure the ileal loop. Several retrospective studies, predominantly from East Asian centers, have reported that the OM is associated with shorter operative time and reduced intraoperative blood loss, while showing no statistically significant increase in short-term postoperative complications [15,16,17,18]. Nevertheless, the available evidence is largely derived from single-region cohorts, and questions remain about its generalizability across healthcare systems and surgical settings.

In addition, many existing studies are limited by retrospective design, small sample sizes and heterogeneity in patient selection, surgical expertise and outcome reporting [10]. Importantly, the absence of statistically significant differences in postoperative morbidity has frequently been interpreted as evidence of comparable safety, despite limited power to detect infrequent but clinically relevant adverse events. Therefore, further institution-level data are needed to provide transparent, context-specific validation of previously reported findings, while carefully distinguishing between statistical significance and clinical relevance.

The present study was designed as a single-center retrospective cohort analysis to compare the OM and the TM of diverting ileostomy in patients undergoing low anterior resection for rectal cancer. The primary objective was to compare short-term postoperative safety outcomes between the OM and TM, while also evaluating potential differences in procedural efficiency, as reflected by operative time and intraoperative blood loss. Secondary objectives included comparison of stoma-related complications, overall postoperative morbidity, anastomotic leakage and length of hospital stay. By reporting our institutional experience from a tertiary center in Türkiye, we aimed to contribute regional validation data and to provide a cautious, practice-oriented interpretation of the OM within the limitations inherent to retrospective observational research.

## 2. Materials and Methods

This retrospective cohort study collected data from patients diagnosed with rectal cancer who underwent surgery with diverting ileostomy between January 2022 and November 2025. The study was conducted in accordance with the World Medical Association’s Helsinki Declaration. Ethical approval was obtained from the local ethics committee (Approval No: 2025/12/05/122, approved on 26 December 2025).

### 2.1. Inclusion and Exclusion Criteria

**Figure 1:**
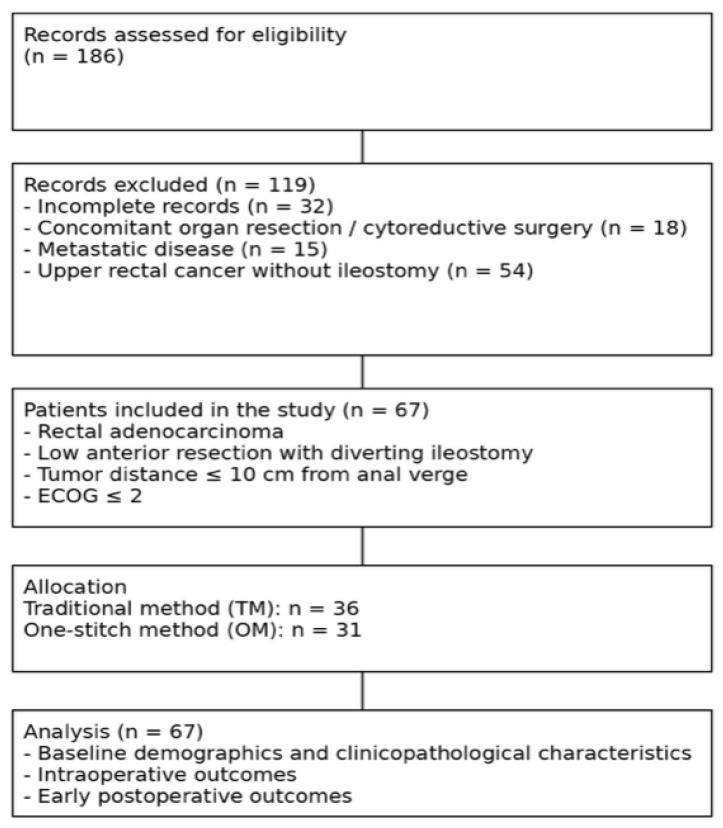
Flowchart of retrospective patient recruitment to compare the outcomes of two methods of protective loop ileostomy during low anterior resection for rectal cancer.

Data from rectal cancer patients who underwent LAR and diverting ileostomy were analyzed (*n* = 186). Patients with incomplete records (*n* = 32), those who underwent other organ resection or cytoreductive surgery (*n* = 18) or MILES procedure (abdominoperineal resection) (*n* = 15) and patients who underwent surgery for upper rectal cancer and did not have an ileostomy (*n* = 54), were all excluded from the study. A total of sixty-seven patients were included in this study (Figure 1).

### 2.2. Data Collection

Clinical, operative and postoperative data were extracted retrospectively from electronic medical records and standardized operative reports. Data extraction was performed by the study investigators using predefined variables. Postoperative complications were identified based on clinical documentation and imaging reports, when available. Stoma-related complications, including skin irritation and mucocutaneous separation, were assessed clinically by surgeons and ostomy care nurses; however, no validated scoring system was applied as a result of the retrospective design of the study.

### 2.3. Definitions

The TNM stage is defined according to the 8th Edition of the American Joint Committee on Cancer (AJCC). Early stoma-related complications were defined as those occurring within 30 days postoperatively, whereas delayed complications were defined as those occurring after 30 days. Early stoma complications for diversion stomas used in middle and lower rectal cancer surgery include stoma retraction, stoma necrosis, stoma skin irritation and mucocutaneous dehiscence, stoma edema, stoma bleeding and stoma infection. Delayed complications include stomal stenosis, stomal prolapse and parastomal hernia. All stoma-related complications and stoma care are diagnosed and treated at our center by qualified ostomy care nurses and clinical physicians.

### 2.4. Protective Ileostomy Surgical Procedure

During the LAR procedure, while creating a conventional diverting ileostomy, the terminal ileal loop was initially identified approximately 30 cm from the ileocecal valve. The terminal ileum was brought out of the abdomen through a small incision in the anterior abdominal wall, through the rectus muscle, which had been properly marked beforehand by ostomy care nurses. Initially, the seromuscular layer of the intestine was sutured to the fascia using 3/0 absorbable Vicryl sutures, with three or four sutures applied. Subsequently, the intestinal lumen was incised longitudinally, and an anastomosis was performed between the intestine and the skin using eight to twelve square knots with 3/0 absorbable Vicryl sutures.

**Figure 2:**
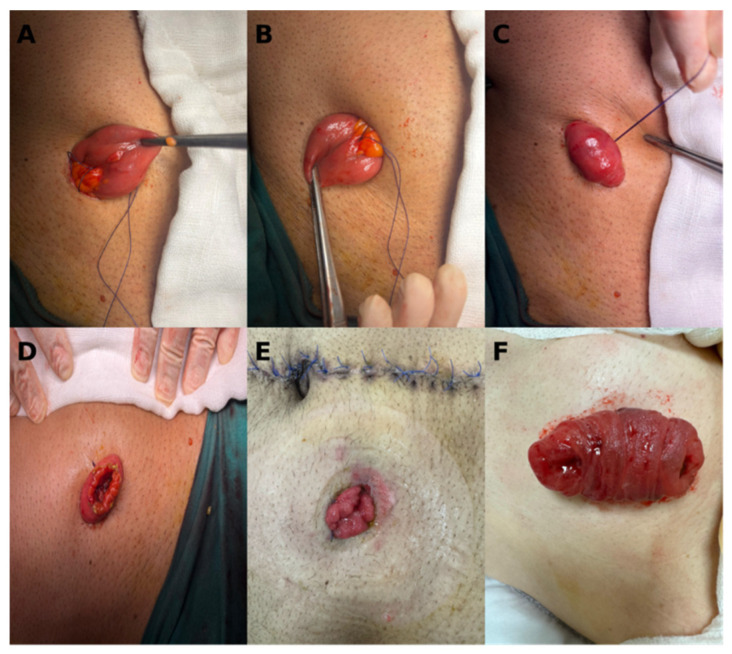
OM technique: step-by-step illustration. (**A**) Placement of a single fixation suture through the mesenteric border of the ileum. (**B**) Passage of the same suture through the skin. (**C**) Stabilization of the ileum after tying the single suture. (**D**) Final intraoperative appearance of the OM. (**E**) Early postoperative appearance. (**F**) Early postoperative appearance (other patient).

The process for determining the location of the terminal ileum in OM ileostomy is the same as in TM. In the OM, after exteriorization of the ileal loop, a single 2/0 absorbable Vicryl suture was passed through subcutaneous tissue, and then both suture sites were passed through the avascular area of the mesentery, followed by passage through the subcutaneous tissue on the other side sides. The suture was tightened to bring the bowel close to the abdominal wall, leaving a 2–3 mm gap to ensure proper perfusion, and tied firmly. The bowel was then opened longitudinally to create the stoma (Figure 2).

The choice of ileostomy technique (OM or TM) was not randomized. The selection was primarily based on surgeon preference and institutional practice during the study period. The OM was gradually adopted in our center, and its use may therefore reflect temporal trends, individual surgeon experience and case selection. No formal protocol mandated the use of one technique over the other, and specific patient-level factors influencing technique choice could not be fully standardized, as a result of the retrospective nature of the study.

### 2.5. Statistical Analysis

The normality of continuous data was assessed using the Kolmogorov-Smirnov and Shapiro-Wilk tests. The assumption of homogeneity of variances was tested using the Levene homogeneity test. Group comparisons were made using the Mann-Whitney U test and the Independent *t*-test, depending on how well the assumptions were met. Descriptive statistics for the variables were summarized as Arithmetic mean ± Standard deviation, Median (IQR, Interquartile Range). Frequencies for categorical variables were given as *n* (%). In comparing categorical variables, the appropriate tests from the Chi-Square test, Fisher Exact test and Fisher-Freeman-Halton Exact test were used, taking into account the number of columns x rows and the expected values in the cells. Statistical analyses of the study were performed using R (version 4.4.1 R Foundation for Statistical Computing) and JASP (version 0.19.0 University of Amsterdam, The Netherlands) software. In all statistical analyses, *p* < 0.05 was interpreted as statistically significant.

Given the limited sample size, no formal propensity score matching or weighting strategy was applied, as such approaches may increase the risk of overfitting and unstable estimates. Multivariable logistic regression was therefore used as an exploratory adjustment method to account for selected clinically relevant covariates. All subgroup analyses were predefined but should be interpreted as exploratory due to limited statistical power.

## 3. Results

A total of 67 patients who underwent low anterior resection with diverting ileostomy for rectal cancer were included in the study. Of these, 31 patients underwent as the OM group and 36 patients underwent as the TM group.

Baseline demographic and clinical characteristics did not differ significantly between the two groups. There were no statistically significant differences in age, sex, body mass index, comorbidities, neoadjuvant treatment rates, pathological T and N stages, or anastomosis level (all *p* > 0.05). The distribution of surgical approach (open versus laparoscopic) also did not differ significantly between the OM and TM groups (*p* = 1.000), indicating no statistically significant differences in operative approach between groups (Table 1).

The operative time was significantly shorter in the OM group (*p* < 0.001; rank-biserial coefficient = 0.56). In addition, intraoperative blood loss was significantly lower in the OM group (*p* < 0.001; coefficient = 0.531). Although the median blood loss was identical between the groups, the interquartile range was narrower in the OM group (100 [50–100] cc) compared with the TM group (100 [100–200] cc), resulting in a statistically significant difference (*p* < 0.001). The median length of hospital stay was numerically longer in the OM group; however, this difference did not reach statistical significance (7.0 [6.0–8.5] vs. 5.5 [5.0–7.0] days, *p* = 0.104).

In both groups, the most common stoma-related complications were skin erythema, parastomal hernia and stricture, with overall complication rates of 19.4% in the OM group and 25.0% in the TM group. However, this difference is not statistically significant (*p* = 0.796; Cramér’s V = 0.032). No significant association was found between neoadjuvant treatment and stoma complications (*p* = 0.268). Anastomotic leakage rates and stages were comparable between the two groups (*p* = 1.000; Cramér’s V = 0.028). No significant difference was found between the groups regarding the anastomosis level (*p* = 0.189; Cramér’s V = 0.411) (Table 2).

Overall postoperative complication rates were similar for both groups (OM: 32.3% vs. TM: 30.6%; *p* = 1.000; Cramér’s V = 0.000). The most common types of complications observed were wound infection and intra-abdominal infection (Table 2).

To minimize potential confounding related to surgical approach, a laparoscopic-only subgroup analysis was performed, including 19 patients in the OM group and 20 in the TM group. Within this subgroup, the OM technique was associated with a significantly shorter operative time (*p* = 0.005). Intraoperative blood loss was also significantly lower in the OM group, with a median of 50 [50–100] cc versus 150 [100–250] cc in the TM group (*p* = 0.001). In contrast, no significant differences were observed between the two groups in postoperative safety outcomes within the laparoscopic-only subgroup. No statistically significant differences were observed between the OM and TM groups (all *p* = 1.000). Likewise, the length of hospital stay showed no significant difference between the two techniques (*p* = 1.000) (Table 3).

In multivariable logistic regression analysis adjusted for surgical approach and neoadjuvant therapy, ileostomy technique was not independently associated with postoperative complications (OR 1.03; 95% CI 0.33–3.20; *p* = 0.965). Laparoscopic surgery was independently associated with a lower likelihood of postoperative complications (OR 0.16; 95% CI 0.05–0.55; *p* = 0.004) (Table 4).

## 4. Discussion

In this single-centerretrospective cohort study, we compared the OM and the TM of diverting ileostomy in patients undergoing low anterior resection for rectal cancer. The principal findings were that the OM technique was associated with a shorter operative time and lower intraoperative blood loss, whereas short-term postoperative outcomes—including stoma-related complications, overall morbidity and anastomotic leakage—did not differ statistically between the two groups. Notably, length of hospital stay was shorter in the TM group.

LAR is one of the primary surgical procedures in the treatment of rectal cancer and one of the most significant complications that may occur after LAR, is anastomotic leakage [5,7,19]. Current clinical studies have shown that creating a diverting ileostomy reduces the risk of anastomotic leakage and, even if anastomotic leakage occurs, reduces the need for emergency surgery [9,19]. Various types of diverting ileostomies have been documented so far [13,14,20]. The classic method requires numerous sutures and is therefore associated with a relatively higher incidence of stoma-related complications [21].

There are several studies in the literature that examine the effectiveness of the OM method. It has been shown that the operating time is shorter in both the initial surgery for rectal cancer and the surgery for ostomy closure [16]. OM has also been associated with shorter operative time inpatients with higher BMI, although reported differences in complication rates and hospital stay should be interpreted cautiously due to study design and sample size [15]. A meta-analysis of 590 patients including six recent studies, showed that patients in the OM group had shorter surgery times, shorter hospital stays and less blood loss during index surgery. The OM group was reported to have fewer stoma-related complications, while no significant difference was observed in overall complications [17]. In a study comparing the outcomes of NOSES (Natural orifice specimen extraction surgery) and OM, ostomy opening times, bowel function recovery times, skin irritation rates and pain scores, were found to be significantly lower in the OM group [18].

From a procedural perspective, the reduction in operative time observed with the OM is clinically plausible and consistent with previous reports. The simplified fixation technique eliminates multiple suturing steps required in the traditional approach, thereby reducing operative complexity. In the context of rectal cancer surgery, where total operative duration commonly exceeds three hours, a reduction of approximately 30–40 min may have practical implications for operating room efficiency, anesthesia exposure, and resource utilization [15,16,17]. However, while statistically significant, the clinical relevance of this time saving should be interpreted within the broader perioperative pathway and institutional context.

Similarly, intraoperative blood loss was lower in the OM group, although median blood loss values were identical between groups and the observed difference was driven primarily by distributional variability, rather than absolute volume. This finding highlights the importance of distinguishing statistical significance from clinical significance, as the magnitude of blood loss reduction observed in this study is unlikely to be clinically meaningful for most patients. Nevertheless, reduced variability in blood loss may reflect more standardized tissue handling and less local trauma with the OM.

With respect to safety outcomes, no statistically significant differences were observed between OM and TM in terms of stoma-related complications, overall postoperative morbidity or anastomotic leakage. Importantly, these findings should not be interpreted as evidence of equivalence or non-inferiority. Given the limited sample size, the study was underpowered to detect small or infrequent adverse events, and the possibility of type II error cannot be excluded. Therefore, the absence of statistically significant differences should be interpreted cautiously and framed as an observation rather than a definitive conclusion regarding comparative safety [10,17].

The shorter length of hospital stay observed in the TM group deserves particular attention, as it contrasts with the procedural efficiency advantages associated with the OM. In the current analysis, this finding cannot be attributed to differences in postoperative complication rates, which were comparable between groups. Rather than speculating on unmeasured factors, this result should be interpreted as a potential early implementation drawback of the OM technique in our institutional setting. Differences in postoperative monitoring practices, discharge thresholds, team familiarity with a newer technique or temporal trends in perioperative care, may have contributed to this observation. From an implementation perspective, this finding underscores that technical simplification does not automatically translate into shorter hospitalization, particularly during early adoption phases [12,22].

Our findings are broadly consistent with previous comparative studies of the OM technique, which have reported shorter operative times and no statistically significant increase in short-term complications. However, most available evidence originates from East Asian centers, often with larger cohorts or propensity score–based designs. The present study does not aim to replicate these methodologies or to establish causal inference; rather, it provides institution-level validation within a different healthcare environment. In this context, our results support the reproducibility of previously reported procedural advantages while highlighting the variability of downstream outcomes such as hospital stay [15,16,17,18].

Several significant limitations of this study should be acknowledged. First, the retrospective single-center design inherently introduces risks of selection bias and residual confounding. The choice of ileostomy technique was influenced by surgeon preference and institutional practice rather than random allocation, and unmeasured confounders may have affected the observed associations. Although multivariable regression analysis was performed, causal inference cannot be established. Second, the relatively small sample size limits statistical power, particularly for infrequent but clinically important outcomes such as anastomotic leakage and severe stoma-related complications. Consequently, the absence of statistically significant differences should not be interpreted as evidence of equivalence or non-inferiority, and the possibility of type II error cannot be excluded. Third, the study focused exclusively on short-term perioperative outcomes and did not include stoma reversal data, long-term stoma-site morbidity (such as parastomal or incisional hernia), or patient-reported outcomes including quality of life, stoma satisfaction and functional impact. As the clinical value of a diverting ileostomy technique extends across the full treatment pathway, this represents a major limitation of the present analysis. Fourth, stoma-related complications were identified based on clinical assessment without the use of validated grading systems, and overall postoperative complications were not systematically classified according to Clavien–Dindo severity. This may limit comparability with other studies and reduce reproducibility. Finally, subgroup analysis restricted to laparoscopic cases should be interpreted as exploratory. The small number of patients limits statistical power and increases susceptibility to false-positive or false-negative findings, particularly in the context of multiple comparisons. These analyses are therefore hypothesis-generating rather than confirmatory.

In summary, this study should be interpreted as a contextual validation of the OM technique rather than a demonstration of superiority or equivalence. The findings suggest that OM offers procedural advantages in terms of operative time, without an apparent increase in short-term complications, but does not confer a clear benefit in hospital stay within our institutional setting. Larger prospective studies incorporating standardized complication grading, stoma reversal outcomes, and patient-centered endpoints are required to define the role of the OM across the full clinical pathway.

## 5. Conclusions

In this single-center retrospective cohort study, the OM of diverting ileostomy was associated with shorter operative time and lower intraoperative blood loss compared with the traditional technique, in patients undergoing low anterior resection for rectal cancer. No statistically significant differences were observed between the two methods with respect to short-term postoperative morbidity, stoma-related complications, or anastomotic leakage; however, the study was underpowered for infrequent safety endpoints, and equivalence cannot be inferred.

Notably, length of hospital stay was shorter in the TM group, indicating that procedural efficiency does not necessarily translate into shorter hospitalization, particularly during early institutional adoption of a newer technique. This finding highlights the importance of evaluating ileostomy techniques within the broader perioperative and implementation context.

Overall, the present study should be interpreted as a regional, institution-level validation of previously reported findings rather than a demonstration of superiority or non-inferiority. While the OM appears to offer procedural advantages, its impact on postoperative recovery and longer-term outcomes remains uncertain. Larger prospective studies incorporating standardized complication grading, stoma reversal outcomes and patient-centered measures are required, in order to define the role of the OM across the full clinical pathway.

## Figures and Tables

**Table 1:** Baseline demographic and clinical characteristics.

Variables	Total (*n* = 67)	OM (*n* = 31)	TM (*n* = 36)	*p*-Value
**Surgical approach**				
Laparoscopic/Open	26/41 (38.8/61.2)	17/14 (54.8/45.2)	20/16 (55.6/44.4)	1.000
**Sex**				
Female/Male	30/37 (44.8/55.2)	14/17 (45.2/54.8)	16/20 (44.4/55.6)	0.953
**Age** **, years**	61.2 ± 10.7	59.2 ± 11.1	63.0 ± 10.2	0.142
**BMI, kg/** **m^2^**	26.3 ± 7.7	27.5 ± 9.9	26.2 ± 4.3	0.787
**Neoadjuvant treatment**	34 (50.7)	18 (58.1)	16 (44.4)	0.266
**Pathologic T stage**				0.699
T0	11 (16.4)	7 (22.6)	4 (11.1)	
T1	3 (4.5)	1 (3.2)	2 (5.6)	
T2	15 (22.4)	6 (19.3)	9 (25.0)	
T3	20 (29.9)	9 (29.0)	11 (30.6)	
T4a	18 (26.9)	8 (25.8)	10 (27.8)	
**Pathologic N stage**				0.886
N0	37 (55.2)	17 (54.8)	20 (55.6)	
N1a	5 (7.5)	3 (9.7)	2 (5.6)	
N1b	10 (14.9)	4 (12.9)	6 (16.7)	
N1c	8 (11.9)	3 (9.7)	5 (13.9)	
N2a	3 (4.5)	1 (3.2)	2 (5.6)	
N2b	4 (6.0)	3 (9.7)	1 (2.8)	
**Pathologic M stage**				1.000
M0	63 (94.0)	29 (93.5)	34 (94.4)	
M1	4 (6.0)	2 (6.5)	2 (5.6)	
**Anastomosis level, cm**	6.5 (1–10)	6 (1–10)	6.7 (2–10)	0.189
**Albumin** **, g** **/dL**	4.13 ± 0.51	4.15 ± 0.51	4.12 ± 0.51	0.807
**Hemoglobin** **, g** **/dL**	12.36 ± 1.63	12.42 ± 1.75	12.30 ± 1.55	0.767
**CEA**	2.17 (IQR 2.61)	2.11 (IQR 2.12)	2.19 (IQR 2.85)	0.432
**ASA score**				0.453
1	1 (1.5)	1 (3.2)	0 (0.0)	
2	31 (46.3)	16 (51.6)	15 (41.7)	
3	34 (50.7)	14 (45.2)	20 (55.6)	
4	1 (1.5)	0 (0.0)	1 (2.8)	
**COPD**	11 (16.4)	7 (22.6)	4 (11.1)	0.206
**Diabetes mellitus**	16 (23.9)	7 (22.6)	9 (25.0)	0.817
**Coronary artery disease**	21 (31.3)	8 (25.8)	13 (36.1)	0.365
**Chronic kidney disease**	4 (6.0)	1 (3.2)	3 (8.3)	0.618

Continuous variables are presented as mean ± standard deviation or median (interquartile range), as appropriate. Categorical variables are presented as number (%). Group comparisons were performed using the independent *t*-test or Mann–Whitney U test for continuous variables and the chi-square test or Fisher’s exact test for categorical variables, as appropriate. *p*-values are provided for descriptive purposes only and should not be interpreted as evidence of equivalence.

**Table 2:** Surgical Outcomes Perioperative and short-term postoperative outcomes in the overall cohort.

Variables	Total (*n* = 67)	OM (*n* = 31)	TM (*n* = 36)	*p*-Value
**Stoma-related complications**				0.796
None	52 (77.6)	25 (80.6)	27 (75.0)	
Bleeding	2 (3.0)	1 (3.2)	1 (2.8)	
Necrosis	0	0	0	
Retraction	1 (1.5)	0 (0.0)	1 (2.8)	
Stricture	5 (7.5)	2 (6.5)	3 (8.3)	
Prolapse	0	0	0	
Parastomal hernia	2 (3.0)	1 (3.2)	1 (2.8)	
Skin irritation	5 (7.5)	2 (6.5)	3 (8.3)	
Mucocutaneous separation	1 (1.5)	0 (0.0)	1 (2.8)	
**Overall postoperative complications**				0.633
None	46 (68.7)	21 (67.7)	25 (69.4)	
Ileus	3 (4.5)	1 (3.2)	2 (5.6)	
Intra-abdominal infection	10 (15.0)	4 (12.8)	6 (16.7)	
Pulmonary complications	3 (4.5)	3 (9.7)	0 (0.0)	
Cardiac complications	1 (1.5)	1 (3.2)	0 (0.0)	
Bleeding	2 (3.0)	0 (0.0)	2 (5.6)	
Acute kidney injury	1 (1.5)	0 (0.0)	1 (2.8)	
**Anastomotic leakage**				0.824
None	59 (88.1)	27 (87.1)	31 (86.1)	
Grade A	6 (9.0)	3 (9.7)	3 (8.3)	
Grade B	1 (1.5)	0 (0.0)	1 (2.8)	
Grade C	2 (3.0)	1 (3.2)	1 (2.8)	
**Length of hospital stay, days**	6.0 (5.0–8.0)	7.0 (6.0–8.5)	5.5 (5.0–7.0)	0.104
**Operative time, min**	207.5 ± 35.0	188.7 ± 30.2	223.6 ± 30.8	<0.001
**Intraoperative blood loss, mL**	100 (100–150)	100 (50–100)	100 (100–200)	<0.001

Continuous variables are presented as mean ± standard deviation or median (interquartile range), as appropriate. Categorical variables are presented as number (%). Group comparisons were performed using the independent *t*-test or Mann–Whitney U test for continuous variables and the chi-square test or Fisher’s exact test for categorical variables, as appropriate. Primary outcomes included operative time and intraoperative blood loss. Secondary outcomes included stoma-related complications, overall postoperative complications, anastomotic leakage, and length of hospital stay. *p*-values reflect unadjusted comparisons and should be interpreted cautiously due to multiple testing and limited sample size.

**Table 3:** Exploratory subgroup analysis of laparoscopic cases.

Variables	OM (*n* = 19)	TM (*n* = 20)	*p*-Value
**Stoma-related complications, *n* (%)**	2 (10.5)	2 (10.0)	1.000
**Overall postoperative complications, *n* (%)**	4 (21.1)	4 (20.0)	1.000
**Anastomotic leakage, *n* (%)**	2 (10.5)	2 (10.0)	1.000
**Length of hospital stay, days**	7.0 (6.0–8.0)	7.0 (6.0–8.0)	1.000
**Operative time, min**	190.4 ± 29.8	233.7 ± 33.6	0.005
**Intraoperative blood loss, mL**	50 (50–100)	150 (100–250)	0.001

Continuous variables are presented as mean ± standard deviation or median (interquartile range), as appropriate. Categorical variables are presented as number (%). Group comparisons were performed using the independent *t*-test or Mann–Whitney U test for continuous variables and the chi-square test or Fisher’s exact test for categorical variables, as appropriate. This subgroup analysis was exploratory and underpowered. Results are presented for descriptive purposes only and should not be interpreted as confirmatory evidence.

**Table 4:** Multivariable logistic regression analysis of factors associated with postoperative complications.

Variables	Odds Ratio (OR)	95% Confidence Interval	*p*-Value
**Ileostomy technique (OM vs. TM)**	1.03	0.33–3.20	0.965
**Surgical approach (Laparoscopic vs. Open)**	0.16	0.05–0.55	0.004
**Neoadjuvant therapy (Yes vs. No)**	2.99	0.89–10.02	0.075

Multivariable logistic regression analysis exploring factors associated with postoperative complications. Covariates were selected based on clinical relevance and event frequency. Odds ratios (ORs) are presented with 95% confidence intervals (CIs). Given the limited sample size, the model should be interpreted as exploratory, and overfitting cannot be excluded. Results should therefore be interpreted cautiously.

## Data Availability

The data presented in this study are available on request from the corresponding author.

## Abbreviations

The following abbreviations are used in this manuscript:

AL	Anastomotic Leakage
ASA	American Society of Anesthesiologists
BMI	Body Mass Index
CAD	Coronary Artery Disease
CEA	Carcinoembryonic Antigen
CKD	Chronic Kidney Disease
COPD	Chronic Obstructive Pulmonary Disease
CI	Confidence Interval
IQR	Interquartile Range
LAR	Low Anterior Resection
LOS	Length of Hospital Stay
OM	One-Stitch Method (of diverting ileostomy)
OR	Odds Ratio
PSM	Propensity Score Matching
TM	Traditional Method (of diverting ileostomy)
